# Anxiety, social responsiveness, and grit among patients with KCNJ11-related neonatal diabetes compared to unaffected siblings

**DOI:** 10.1007/s00592-025-02598-w

**Published:** 2026-01-27

**Authors:** Jui M. Desai, Lisa R. Letourneau-Freiberg, Kristen E. Wroblewski, Megan N. Scott, Michael E. Msall, Siri Atma W. Greeley

**Affiliations:** 1 Kovler Diabetes Center, Department of Medicine, University of Chicago, Chicago, IL 60637 USA; 2 Department of Public Health Sciences, University of Chicago, Chicago, IL 60637 USA; 3 The Pritzker Department of Psychiatry and Behavioral Health, Ann & Robert H. Lurie Children’s Hospital of Chicago, Chicago, IL 60611 USA; 4 Department of Pediatrics, University of Chicago, Chicago, IL 60637 USA

**Keywords:** Diabetes mellitus, Potassium channels, Brain, Anxiety, Resilience, Genetics

## Abstract

**Aims:**

Neonatal diabetes mellitus (NDM) occurs before 6–12 months of age and is commonly caused by activating mutations in *KCNJ11* (*KCNJ11*-NDM) or *ABCC8*. Because of brain expression of these mutant ATP-dependent potassium channels, a spectrum of divergent neurodevelopmental difficulties have been described, including developmental delay, epilepsy, and neonatal diabetes (DEND). However, information on anxiety, social responsiveness, and grit is limited.

**Methods:**

Individuals with *KCNJ11*-NDM (*N* = 12) and their unaffected siblings (*N* = 12) were recruited through the University of Chicago Monogenic Diabetes Registry and participants or their parent/caregiver completedthe Screen for Adult/Child Anxiety Related Disorder (SCAARED/SCARED), the Social Responsiveness Scale, Second Edition (SRS-2), and the Grit Scale.

**Results:**

Mean SRS-2 scores were significantly different between *KCNJ11*-NDM and sibling controls (*P* = <0.001 ), with 7/10 affected participants, and 0 /11 siblings, having scores suggestive of autism spectrum disorder (ASD). Differences in anxiety (*P* = 0.69) and grit (*P* = 0.46) were not significant when compared to sibling controls; however, 58% (7/12) of *KCNJ11*-NDM participants and 40% (4/10) of sibling controls had scores indicating an anxiety disorder by either self- or parent-report.

**Conclusions:**

Our results agree with previous studies suggesting significant difficulties with social functioning in *KCNJ11*-NDM, with 7/10 participants having scores suggestive of ASD, strongly reinforcing the need for early neurodevelopmental screening to allow for prompt support. Our report adds to the knowledge of this population in finding robust grit scores but with a high level of anxiety in both *KCNJ11-*NDM and unaffected siblings. Although families affected by *KCNJ11*-NDM may have a high risk of anxiety disorders, it is encouraging that affected and unaffected children exhibit robust self-resiliency that will help support functioning through the challenges of life. Study of additional individuals will help to clarify specific challenges, long-term outcomes, and best approaches for monitoring and support.

**Supplementary Information:**

The online version contains supplementary material available at 10.1007/s00592-025-02598-w.

## Introduction

Neonatal diabetes mellitus (NDM) is defined as diabetes with onset before 6–12 months of age and occurs in approximately 1 in 100,000 live births [1, 2]. Among individuals diagnosed with NDM, nearly half are caused by heterozygous activating mutations in the ATP-dependent Potassium (KATP) channel genes: *KCNJ11* or *ABCC8* and can usually be treated successfully with oral sulfonylureas rather than insulin injections [3–7]. In addition to hyperglycemia, affected individuals also exhibit divergent neurodevelopmental features which arise from the presence of altered KATP channels in the brain. Although many reports have suggested that sulfonylurea treatment may improve neurodevelopmental functioning, the degree to which improvement is possible, and the factors on which it may depend remain uncertain [8–12].

Several studies have shown that more severe neurological phenotypes in KATP-NDM are often related to specific mutations: DEND (developmental delay, epilepsy, and neonatal diabetes) with certain rare mutations, and iDEND (intermediate DEND) with the relatively common V59M mutation, for example. However, other less obvious features appear to be common even in those with mutations not as definitively associated with a neurological phenotype, such as learning disorders and attention-deficit/hyperactivity disorder (ADHD) [13, 14]. Additional careful investigation by our group and others using validated neuropsychological assessments has revealed that affected individuals frequently exhibit a wide range of neurodevelopmental difficulties including decreased social interactions and flexibility/adaptability, increased atypicality, and the presence of psychiatric disorders [15–17]. While several of these studies have noted concerns about social functioning on a variety of measures, it remains unclear how often individuals with *KCNJ11* mutations have impairment at a level seen in those with autism spectrum disorders (ASD), which is an additional co-morbid diagnosis among some within this population. Lastly, anxiety has rarely been directly assessed and to our knowledge grit (i.e. perseverance in settings of adversity) has not been measured, but both are important aspects of functioning within individuals with any type of chronic condition and/or neuroatypicality. Studies on anxiety in other chronic conditions such as type 1 diabetes suggest the importance of early identification and management to optimize long term well-being [18, 19].

In this study, we assessed self- and/or parent/caregiver-completed surveys on anxiety, grit, and social responsiveness in participants with *KCNJ11*-NDM and their unaffected sibling controls. We hypothesized that participants with *KCNJ11*-NDM would score worse on measures of social responsiveness, report more anxiety, and report less grit than their unaffected siblings.

## Methods

### Compliance with ethical standards

**Fig. 1:**
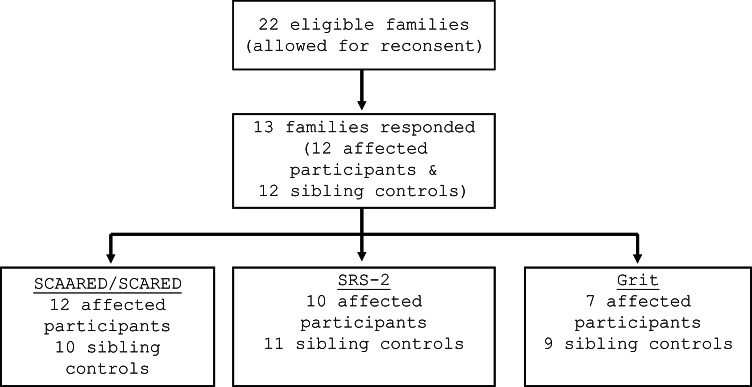
Participant Recruitment

Participants with *KCNJ11*-NDM and their unaffected siblings were recruited and consented for this study through the University of Chicago Monogenic Diabetes Registry (http://monogenicdiabetes.uchicago.edu/registry/). This study was performed in line with the principles of the Declaration of Helsinki. Approval was granted by the Ethics Committee of University of Chicago (IRB 6858, 15617B, 16935B) (Fig. 1). The Registry gathers extensive longitudinal data about monogenic diabetes participants, such as diabetes diagnosis and treatment data, other medical problems, diabetes genetic testing results, and family history information [20].

Three standardized behavioral and neuropsychological surveys were given to each participant and/or parent/caregiver: the Screen for Adult/Child Anxiety Related Disorder (SCAARED/SCARED) [21], the Social Responsiveness Scale, Second Edition (SRS-2) [22], and Grit Scale [23, 24]. SCAARED/SCARED generates a total score as well as scores for following subscores: Panic Disorder or Significant Somatic Symptoms (PA/SO), Generalized Anxiety Disorder (GAD), Separation Anxiety Disorder (SEP), Social Phobic Disorder (SOC), Significant School Avoidance Symptoms (SCH). The scores range from 0 to 62, with a score ≥25 indicating the presence of an Anxiety Disorder for child reports (child self-report or parent-report) and a score ≥23 indicating the presence of an Anxiety Disorder for adult self-report.

We chose the SRS-2 for describing a total social responsiveness score, as well as five content areas of social functioning including Social Awareness (AWR), Cognition (COG), Communication (COM), Motivation (MOT), and Restricted Interests and Repetitive Behavior (RRB) for children, adolescents, and adults. The total score and other scores are reported as T-scores from a range of 0 to 90. A total T-score ≤59 is within normal limits, 60-65 is mild range, 66-75 is moderate range, and ≥76 is considered severe range.

The SRS-2 and SCAARED/SCARED generated scaled scores that could be analyzed despite differences in participant age, enabling a greater cohort of participants. In situations where both a self- and parent-reported form were completed, the self-report was used for analysis, unless otherwise indicated. 

The Grit Scale measures the extent to which individuals can maintain focus and interest and persevere in obtaining long-term goals. A high grit score indicates that the individual is resilient, persistent, and able to overcome obstacles to successfully achieve their goals [23]. Grit scores range from 1 to 5, with 5 being the maximum score and indicating the highest level of grit.

To compare the three measures, nonparametric analyses were performed using the Mann-Whitney U-test for group comparisons with Stata 19 (StataCorp LLC, College Station, TX) for statistical analysis. Results are expressed as mean ± standard deviation (SD) unless indicated, and group differences were considered significant if *P* < 0.05.

## Results

### Participants

Twelve participants with *KCNJ11*-NDM and twelve unaffected sibling controls participated in this study. The median age at assessment was 13.8 years for *KCNJ11*-NDM participants and 14.1 years for sibling controls (Table 1). Comparison of the mutations of affected participants in the study versus affected individuals not in the study did not reveal any distinct differences that may have preferentially influenced participation in the study. See Figures S1-S2 in the Supplementary Material for comprehensive demographic information as well as comparisons of mutations.

**Table 1:** Study participant demographics: data are given as medians with interquartile range, and minimum, maximum in parenthesis

	KCNJ11-related neonatal diabetes	Sibling Controls
n	12	12
Age at assessment (years)	13.8 (10.6–22.65, 5.4, 26)	14.05 (10.4–15.4, 7, 20.1)
Women (%)	7 *(58)*	8 *(67)*
Gene Mutation Causative variant in KCNJ11	12 KCNJ11 7 V59M 2 R201H 1 R201C 1 A174G 1 G53D	Not applicable
Age first started taking sulfonylureas (years)	1.4 (1.05–13.36, 0.15, 14) (0.23–9.9, 0.02, 14)	Not applicable

### SCAARED/SCARED results

From the SCAARED/SCARED, the mean total anxiety score was 25.3 (±16.0) for *KCNJ11*-NDM participants and 22.7 (± 12.2) for unaffected sibling controls. Statistical analyses revealed no significant differences in the mean total anxiety scores (*P* = 0.69), as well as all subscores, in *KCNJ11*-NDM versus unaffected sibling participants (Table 2).

**Table 2:** Anxiety related disorders results (SCARED/SCAARED): table legend: PA/SO (Panic disorder or significant somatic Symptoms), GA (Generalized anxiety disorder), SEP (Separation anxiety disorder), SOC (Social phobic disorder), SCH (Significant school avoidance Symptoms). U Value was calculated treating the KCNJ11-related neonatal diabetes group as group 1

	KCNJ11-related neonatal diabetes		Sibling Controls		Analysis	
	n	Mean ±SED	n	Mean ± SED	P-value	U Value
Total Score	12	25.3 ± 16.0	10	22.7 ± 12.2	0.69	66
PA/SO	12	7.8 ± 8.1	10	4.6 ± 4.9	0.37	73.5
GA	12	8.2 ± 6.2	10	6.5 ± 5.4	0.49	70.5
SEP	12	4.3 ± 3.3	10	4.9 ± 2.5	0.82	56.5
SOC	12	4.1 ± 2.9	10	5.6 ± 3.6	0.32	45.0
SCH	7	1.9 ± 2.8	9	1.2 ± 1.4	0.87	33

Despite there being no statistically significant differences, it is important to note that according to SCAARED/SCARED clinical categorization, seven out of twelve participants (58%) with *KCNJ11*-NDM and four out of ten sibling controls (40%) met criteria for an anxiety disorder by either self- or parent-report. Of note, one affected participant met criteria for an anxiety disorder on the parent-report form but did not meet criteria for an anxiety disorder on the self-report form, and one sibling control met criteria for an anxiety disorder on the self-report form but did not meet criteria for anxiety on the parent-report form.

### SRS-2 results

**Fig. 2:**
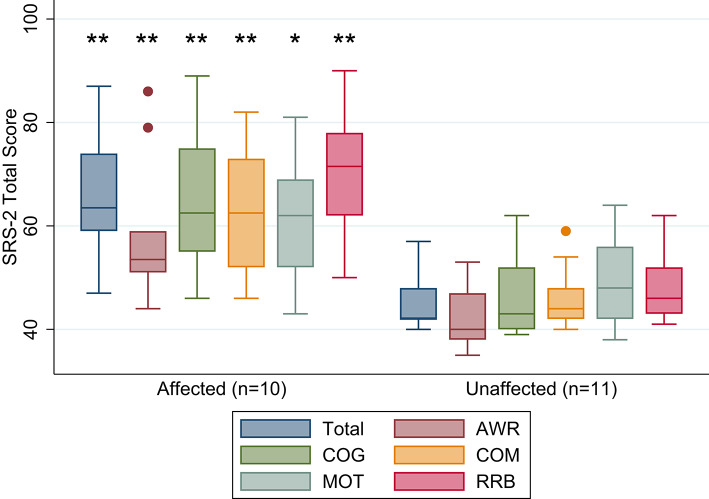
Social Responsiveness Scale-2 Scores (** *P* < 0.01; *P < 0.05): Affected participants exhibit significantly worse total social responsiveness, as well as worse social awareness, cognition, communication, motivation, and repetitive and restrictive behavior. However, despite the statistical significance, the affected participants exhibited similar motivation to unaffected participants (*P* = 0.49)

The mean total SRS-2 score was 66.2 (± 13.1) for *KCNJ11*-NDM and 45.9 (± 6.1) for unaffected siblings. *KCNJ11*-NDM participants displayed significantly worse SRS-2 mean scores than unaffected sibling controls (*P* < 0.001) as well as significantly worse subscores. The association appeared to be less strong for the Motivation subscore (*P* = 0.029) (Fig. 2).

Additional analysis of the clinical categorization revealed that 7 out of 10 *KCNJ11*-NDM participants (70%) had scores within a range consistent with mild (*n* = 2), moderate (*n* = 3), or severe (*n* = 2) associations with autism spectrum disorder while 0 out of 11 sibling controls (0%) had scores within a range consistent with autism spectrum disorders (Table 3). There was no clear association with mutation with the severity category.

**Table 3:** Social responsiveness Scale-2 total score. Displays the participants within ranges associated with autism spectrum disorder and respective causative variants

SRS-2 Total Score Discussion	KCNJ11-NDM	Causative Variant	Sibling Controls
n	10		11
Within normal limits	3 (3F)	V59M, R201C, R201H	11 (8F 3M)
Mild range	2 (1 F 1 M)	V59M, R201H	0
Moderate range	3 (1F 2M)	V59M, A174G, G53D	0
Severe range	2 (2M)	V59M, V59M	2 (2 F 0 M)

*Within normal limits* (generally not associated with autism spectrum disorders); *Mild range* (indicate deficiencies in reciprocal social behavior that are clinically significant and may lead to mild to moderate interference with everyday social interactions); *Moderate range* (indicate deficiencies in reciprocal social behavior that are clinically significant and may lead to substantial interference with everyday social interactions); *Severe range* (indicate deficiencies in reciprocal social behavior that are clinically significant and may lead to severe interference with everyday social interactions)

### Grit results

Grit generates a single grit score, which was 3.3 ± 0.5 for affected participants and 3.4 ± 0.4 for unaffected participants. Only 7 affected participants completed the Grit Scale. Statistical analysis revealed no significant differences in grit scores (*P* = 0.46) (Table 4). The average grit scores reflect the mean on items for the whole group.

**Table 4:** Neurodevelopmental findings – Grit. U value calculated treating the KCNJ11-related neonatal diabetes group as group 1

	KCNJ11-related neonatal diabetes		Sibling Controls		Analysis	
	n	Mean ± SED	n	Mean ± SED	P-value	U value
Grit	7	3.3 ± 0.5	9	3.4 ± 0.4	0.46	24.5

## Discussion

In this study, we found that individuals with *KCNJ11*-NDM had significantly worse social responsiveness compared to their sibling controls, indicative of ASD. There was no group difference in assessment of anxiety or grit; however, 7 out of 12 with *KCNJ11-*NDM and 4 out of 10 unaffected siblings had scores indicative of an anxiety disorder by self- or parent-report.

Similar to previous studies by our group and others [16, 25, 26], showing a variety of struggles including social dysfunction, individuals with *KCNJ11*-NDM exhibited significantly worse social responsiveness in both total scores, as well as on all five subscales including Social Awareness, Cognition, Communication, Motivation, and Restricted Interests and Repetitive Behavior. The association appeared to be less strong for the Motivation subscale. Out of the five subscales, Motivation is the only intrinsic measure, leading us to speculate that participants with *KCNJ11-*NDM may exhibit an internal sense of motivation, which can be connected to grit and self-resiliency.

Although the SRS-2 is not meant for diagnosis of ASD, our results support a strong association between *KCNJ11-*NDM and autism spectrum disorder [27]. This finding reinforces the existing but limited data on the relation between these two conditions [26] and encourages providers to explore existing interventions in improving social competencies in people with features of autism spectrum disorders [28]. We had a high participation rate of participants with V59M causative variant, but unexpectedly, they exhibited a range of scores associated with autism spectrum disorder, including one without significant social concerns. This participant scored in the ‘normal’ range on their parent-completed SRS-2; there is no clear hypothesis as to why this participant would score better than other participants with V59M. One additional V59M participant scored in the 'mild' range. This participant was serendipitously exposed to sulfonylureas in utero, as has been described previously [29], which may explain why they scored better than other participants with V59M. Furthermore, a few individuals with mutations associated with a milder phenotype (such as R201H) had scores within a range concerning for autism spectrum disorder. This indicates that all affected individuals should undergo comprehensive evaluation regardless of specific mutation. Although siblings had similar anxiety scores as affected participants, no siblings had SRS-2 scores suggestive of ASD, whereas 70% of the *KCNJ11*-NDM individuals had scores suggestive of ASD. This large difference between affected participants and their unaffected siblings suggests that most of these families may not have a high background genetic risk for ASD, as may be the case in the general population [30], and the affected participants are likely to have “monogenic” ASD due to their *KCNJ11* variant.

Although individuals with *KCNJ11*-NDM did not exhibit significantly worse anxiety, both affected participants (7/12, 58%) and their sibling controls (4/10, 40%) scored in a range indicative of an anxiety disorder by self- or parent-report. These rates are higher than the overall lifetime prevalence of any anxiety disorder among adolescents in the US of 31.9% [31]. When one member of the family is impacted by a chronic disease such as T1DM, there can be increased psychological impact and anxiety on other unaffected members [32]. This notable finding highlights the importance of assessing anxiety levels not only with participants with *KCNJ11*-NDM, but also their siblings and other close family members.

Grit results were not significantly different when comparing individuals with *KCNJ11*-NDM to their sibling controls. The lack of significant differences of the Grit scores, which measure more intrinsic levels of personality, lead us to postulate that participants with *KCNJ11*-NDM exhibit intrinsic grit.

This study provides a greater understanding into the neurodevelopment difficulties individuals with *KCNJ11*-NDM may experience. These results can help improve patient clinical awareness: early screening for specific neurodevelopment difficulties such as social responsiveness can facilitate early intervention for individuals to help combat potential difficulties. To our knowledge, this is the first study investigating self- or parent/caregiver-reported grit in children and early adults with *KCNJ11*-NDM and to compare anxiety between them and their unaffected sibling controls. Additionally, our findings regarding heighted anxiety levels at the familial level encourage providers to advocate for greater support for families affected by *KCNJ11-*NDM.

We recognize that our sample size for this study is not very large, making it difficult to draw extensive conclusions about these neurodevelopment findings as representative of the *KCNJ11*-NDM community. However, even small samples can be meaningful in rare disease research such as neonatal diabetes. Additionally, questionnaires were completed by the participants and/or their family members instead of an external individual. This allows for potential personal biases or perceptions to affect the accuracy of the questionnaire results.

## Conclusions

In summary, individuals with *KCNJ11-*NDM have significantly worse social responsiveness than their sibling controls. Anxiety rates and grit are similar in individuals with *KCNJ11*-NDM compared to their sibling controls. Thus, it would be beneficial to monitor and check children with *KCNJ11-*NDM for social responsiveness challenges and anxiety challenges to obtain early support. Our findings suggest that unaffected siblings of individuals with *KCNJ11*-NDM may also benefit from an assessment for anxiety. Grit does not appear to be as different; individuals with *KCNJ11-*NDM exhibit self-resiliency and grit at rates similar to sibling controls, which should support them achieving their individual goals in life.

## Supplementary Information

Below is the link to the electronic supplementary material.

Supplementary Material 1

## Data Availability

The datasets generated during the current study are available from the corresponding author on reasonable request.
