# Supplementary material for: Anxiety, social responsiveness, and grit among patients with KCNJ11-related neonatal diabetes compared to unaffected siblings
Source: Acta Diabetol. 2026 Jan 27;63(4):621–7. doi: 10.1007/s00592-025-02598-w (PMC13092531; doi:10.1007/s00592-025-02598-w)
Supplement: Supplementary file 1 — Supplementary Material 1 [file 592_2025_2598_MOESM1_ESM.docx]

**Anxiety, Social Responsiveness, and Grit among Patients with KCNJ11-Related Neonatal Diabetes Compared to Unaffected Siblings**

Desai et al

Supplementary Figures

S1: Demographics by Sex: Fisher’s exact p > 0.99

|  | Biological Sex | |  |
| --- | --- | --- | --- |
| Case | **Women** | **Men** | **Total** |
| Unaffected  (%) | 8  66.67 | 4  33.33 | 12  100.00 |
| Affected  (%) | 7  58.33 | 5  41.67 | 12  100.00 |
| Total  (%) | 15  62.5 | 9  37.5 | 24  100.00 |

S2: Demographics of Individuals who did not Participate in Study

|  | KCNJ11-related neonatal diabetes |
| --- | --- |
| n | 10 |
| Women (%) | 3 *(30)* |
| Gene Mutation  Causative variant in KCNJ11 | 10 KCNJ11  1 V59M  5 R201H  1 K170N  1 R201C  1 R50P  1 R34C |
